# A Music Interaction System Based on the Emotional Recognition of Older Adults in Smart Homes: Cross-Sectional Experimental Study

**DOI:** 10.2196/77218

**Published:** 2026-02-10

**Authors:** Chengmin Zhou, Yuxuan Luo, Wenjing Zhan, Ting Huang, Jake Kaner

**Affiliations:** 1 College of Furnishings and Industrial Design, Nanjing Forestry University, No. 159, Longpan Road, Xuanwu District, Nanjing, Jiangsu Province, 210037, China, 86 13851562125; 2 Jiangsu Co-Innovation Center of Efficient Processing and Utilization of Forest, Nanjing, China; 3 Faculty of Natural, Mathematical and Engineering Sciences, King's College London, London, United Kingdom; 4 School of Art and Design, Nottingham Trent University, Nottingham, United Kingdom

**Keywords:** music emotion model, electroencephalogram, geriatric HCI, age-friendly, model building

## Abstract

**Background:**

The advent of the Smart Home 3.0 era imposes higher technical requirements for the construction of the home ecosystem. Music is an effective means for humans to regulate their emotions. The emergence of multimodal technology facilitates the use of music for emotional regulation by older adults in their home environment. Therefore, it is necessary to carry out further research on the emotional issues of the music interaction system in smart homes that are targeted at older adult users.

**Objective:**

This study aimed to establish the mapping relationship between music parameters and emotional states based on the Valence-Arousal emotional model and through the analysis of electroencephalogram (EEG) signals. In addition, a novel Multimodal Fusion-Adaptive Wellness Interaction model was constructed to match the most suitable music and meet the emotional needs of older adult users.

**Methods:**

A total of 68 older adult participants aged between 60 and 75 years were recruited. Four groups of music stimulus materials were formulated to conduct an experimental study on the relationship between the emotions of older adult participants and music parameters. Finally, the Multimodal Fusion-Adaptive Wellness Interaction model was constructed. With the recreational sofa as the main body, multimodal, emotional intelligent adaptive interaction was realized.

**Results:**

During the experiment, approximately 68,970,528 EEG signal data were collected. After analyzing the EEG bands and extracting the event-related potential data, the following results were obtained: For P200, the music stimulus materials showed significant differences and activation in the left parietal and occipital lobe regions (P7, P3, O1, O2; *P*<.001). For N400, there were significant differences and activation in the prefrontal lobe region due to the music stimulus materials (O1, CZ, O2; *P*<.001). For P300, the music stimulus materials led to significant differences and activation in the occipital lobe region and the central line region of the temporal area (F7, FPZ, FZ, F8; *P*<.001).

**Conclusions:**

Older adult users respond in a way that the A1 stimulus material, namely the music material with positive valence and high arousal (+V + A), can soothe the emotional state of older adult users. This provides a theoretical basis and evidence for the music interaction regulation module in smart home systems.

## Introduction

The concept of the Smart Home has continually evolved over the past few decades. It was first introduced in the United States in 1984, when United Technologies Building presented the “Intelligent Building” with integrated and information-based building equipment [1]. With the advancement of Internet of Things technology, smart homes entered the 2.0 era, characterized by interconnectivity and remote control [2]. The advent of the Smart Home 3.0 era imposes higher technical requirements for the construction of home scenarios and user experience, characterized primarily by scenario-based synergy and context awareness [3]. During this evolution, human-computer interaction (HCI) modes have evolved from single-channel physical interfaces, such as buttons in traditional smart home devices, to multimodal systems that integrate multiple input modalities, including voice, gesture, and eye movement [4]. This low-burden multimodal interaction not only reduces operational difficulty but also facilitates emotional companionship and psychological comfort. As a key potential user group for smart homes, older adults warrant particular attention regarding their growing needs for both physical and emotional support. Their acceptance and use of smart home devices exhibit significant heterogeneity, with increasing engagement particularly in voice-activated and entertainment devices [5]. Emotional interaction is becoming a mainstream adaptive strategy for smart homes in domestic environments, garnering increasing scholarly focus on the emotional integration and psychological adaptation of older adults within their home settings [6]. Consequently, this project aims to explore the integration of multimodal information to achieve adaptive interaction within a smart furniture system. By focusing on behavioral and emotional interaction, it seeks to provide older adult users with an optimized experience that promotes both leisure and emotional well-being.

Among various interaction modalities, music is widely recognized as an effective medium for emotional regulation and for supporting cognitive health for older adults [7]. As a complex auditory input, music plays a crucial role in influencing emotions within the home environment [8,9]. For the older adult population, music serves as a noninvasive means of emotional regulation, helping to alleviate negative emotions such as loneliness, anxiety, and depression, while also enhancing quality of life and overall home-based well-being by evoking positive emotional experiences [10]. Previous studies have demonstrated that music listening can effectively promote social engagement and emotional health among older adults and even improve cognitive function and memory to some extent [11]. Moreover, with the accelerating process of population aging, the dependence and engagement of older adults with music continue to increase. For example, in the United States, more than 50% of older adults report listening to music daily [12]. This indicates that music has gradually become an essential part of daily recreation for older adults and is regarded as an effective approach for emotional regulation and maintaining psychological well-being [13]. Therefore, the design and application of music-based interactions in smart home environments for older adult users hold substantial research significance.

In exploring music’s role in emotional regulation mechanisms, studies have found that music intervention can promote hippocampal neurogenesis and improve emotional states [14]. At the same time, music primarily regulates users’ emotions by influencing neuronal activity in the brain [15]. Therefore, using physiological measurement devices to examine human emotional changes offers multiple advantages [16], as these signals can continuously and objectively reflect users’ behavioral and experiential information. By combining physiological measurements with subjective emotional assessments, researchers can more accurately reveal the mapping relationship between music and emotional states [17].

Compared with subjective evaluations, physiological measurements provide a more objective and comprehensive description of emotional changes [18]. Consequently, neuroscience research, particularly studies using electroencephalography, a noninvasive neurophysiological signal, has been widely applied to emotion recognition and regulation in recent years [19]. From a neural mechanism perspective, emotional processing in the human brain relies on complex neural networks. The amygdala is responsible for evaluating the emotional significance of stimuli, whereas the insular cortex modulates emotional responses [20]. Electroencephalogram (EEG) studies related to emotion have sought to clarify the roles of different frequency bands and brain regions in emotional processing [21,22]. Research has shown that emotional content can amplify certain event-related potentials (ERPs) in EEG signals [23], while hemispheric asymmetry in theta power reflects emotional valence [24]. Moreover, variations in EEG activity in the frontal and parietal lobes, including α, β, θ/β, and σ/β ratios, are associated with individual differences in emotional regulation [25]. The integration of musical stimuli and EEG signals has gradually evolved into the field of music neuroscience [26]. For instance, studies combine analyses of musical elements with EEG data to investigate music perception and training effects [27]. Recording motor-related EEG responses evoked by music has been regarded as a key approach to understanding emotional experiences [28]. By analyzing the neural activity patterns elicited by music through EEG, researchers can gain deeper insights into the mechanisms underlying older adults’ emotional experiences induced by music.

The development of multimodal technologies has enabled smart home systems to capture the relationship between users’ emotions and music within home environments with greater accuracy. On the one hand, multimodal perception methods such as speech recognition, facial expression analysis, and physiological signal monitoring allow for a more comprehensive understanding of the emotional states of older adults in home settings [29]. Moreover, studies have demonstrated that different musical melodies and rhythms can elicit distinct emotional responses in users [30-33]. Integrating music with smart home technologies can thus serve as an essential multimodal pathway for emotional regulation, providing older adult users with low-burden and continuous emotional regulation support through a closed loop of perception, feedback, and intervention.

With the support of multimodal technologies, smart homes can serve as an important platform for music-assisted emotional regulation. In fact, previous studies have already begun to explore the potential of integrating smart home technologies with music-based emotion regulation. Currently, emotional regulation in smart homes is mainly achieved through 2 approaches. The first involves contact-based acquisition of physiological signals to recognize and regulate emotions. For example, Wagner et al [34] extracted 3 types of features from heart rate, electrodermal activity, electromyography, and respiration signals and used multiple classifiers for emotion recognition with promising results. The second approach relies on computer vision analysis, using cameras to capture users’ facial expressions for emotion recognition and feedback. For instance, Yu et al [35] designed a prototype of smart furniture called the “Magic Mirror Table,” which can infer users’ emotional states. It captures facial expressions through a camera, performs detailed analysis, and then plays appropriate sentences or music to influence users’ emotions. Compared with other emotion recognition techniques, emotion regulation technologies applied in smart home environments have the advantage of passively and continuously collecting multimodal data in natural living contexts [36], thereby providing a more authentic reflection of individual emotional changes.

In the field of multimodal interaction, previous studies have explored various approaches to integrating visual, auditory, motion, and physiological signals to enhance emotion recognition and HCI performance. In the context of smart homes, multimodality enables systems to respond to user needs in a more natural and integrated manner [37]. Some studies have proposed multimodal interaction systems based on 3 different modalities, eye blinking, voice, and touch, to allow individuals with limited mobility to control smart home devices [38]. Other researchers have introduced multimodal interfaces designed to support independent living for older adults in smart home environments [39]. However, in domestic settings, key challenges for multimodal interaction include user diversity and the dynamic nature of interaction contexts [40,41]. Existing studies have primarily focused on improving home interaction by adapting to users and environmental conditions [42-44]. Current mainstream control modes in smart homes include voice and gesture interaction. Related research has also proposed a trajectory recognition interaction method based on vision, acceleration, and dynamic gestures, verifying its feasibility for integration into multimodal and adaptive smart home systems [45]. In terms of emotional regulation, multimodal interaction research has been applied to distraction detection, emotion recognition, and stress-level assessment [46]. Other scholars have investigated the relationship between multimodal fusion of emotional information and interactive behaviors. Their studies used emotional color as the theoretical basis for affective computing in HCI and applied neural network algorithms to datasets, ultimately developing an efficient multimodal affective interaction recognition method [47].

In this study, we expect to explore the relationship between different types of emotional music and the EEG changes of older adult users and derive the degree of stimulation of brain waves by other music genres, which will further perfect the emotion recognition linkage module of the smart home with the sofa as the carrier. The main contributions of this work include the following:

1. Innovative construction of the Multimodal Fusion-Adaptive Wellness Interaction (MF-AWI) model. The MF-AWI model theory can be disassembled into the Multimodal Fusion-Adaptive Wellness model. The main modules can be divided into a posture recognition module, an emotion recognition module, and a wellness HCI module. The theoretical approach guides the realization of the MF-AWI Multimodal Fusion Adaptive Wellness System. This article focuses on the emotion recognition module, relying on the theoretical basis, to achieve the real-time state of the recreation sofa on the older adult users’ real-time state recognition, to make the real-time feedback of the recreation adjustment within the scope of the music adjustment, to meet the personalized needs of different older adult users in different scenarios.
2. In the module on emotional state recognition for older adult users, 4 music genres were classified based on the theory of the Valence-Arousal (VA) emotion model, and a control group was set up to explore the role of music genres in the emotional regulation of older adult users. Through the EEG physiological signals, the best music genres for older adult users in different scenarios were identified.

## Methods

### MF-AWI Model

**Figure 1.**
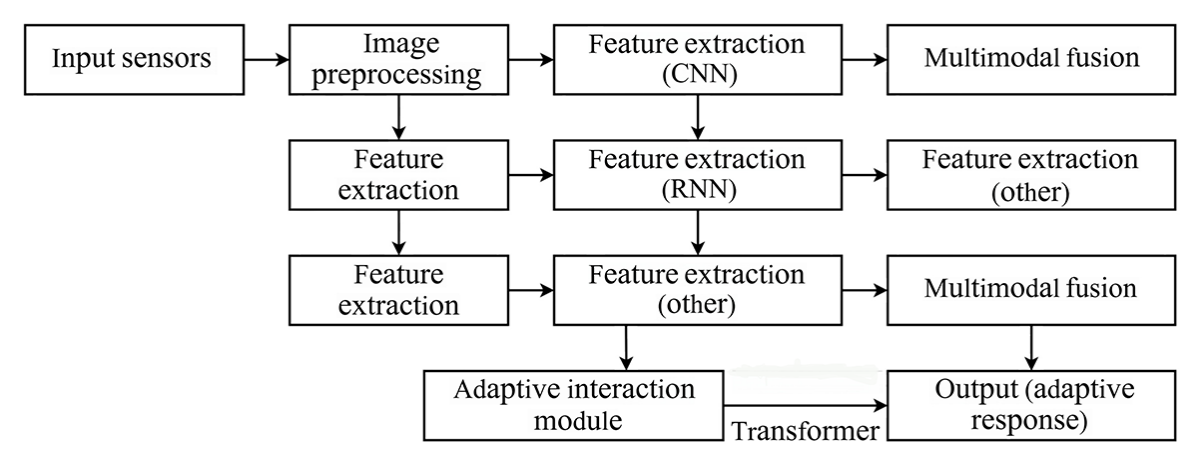
The Multimodal Fusion-Adaptive Wellness Interaction model responds to process iterations. CNN: convolutional neural network; RNN: recurrent neural network.

The MF-AWI model theory can be decomposed into the MF-AWI model, which relies on the foundation of Multimodal Fusion theory to dynamically identify the target participant, locate the target participant, and use the MF-AWI model. The core of the MF-AWI model is based on the concept of multimodal fusion, that is, the integration of information from multiple sources or modalities to gain a more complete understanding of the target participant. In the context of the MF-AWI model, these modalities are poses and emotions that are used to identify the target participant and decompose the target state into its components, as shown in the recognition process in Figure 1.

The unified representations will be fed into the Wellness HCI module, which will use the pretrained Transformer model to generate adaptive feedback. The Wellness HCI module can recognize different gestalt states and emotional states of the older adult users, enabling the system to provide appropriate adaptive feedback to different older adult users’ inputs. For example, if the user indicates that they want to go to a resting state based on their posture and emotional state, the Wellness HCI module can suggest that the wellness sofa adjust the backrest angle to give the older user a more comfortable position, or play soothing music to create a more relaxing environment. Similarly, if the older adult users appear to have negative emotions such as stress or anxiety, the Wellness HCI module can suggest relaxation exercises or provide calming music to help the user relax. The output of the wellness HCI module will be used to control the different module functions of the wellness sofa, including the adjustment of functional modules such as angles and music. This process can be repeated to provide a personalized and adaptive interactive experience for the older adult user on the wellness sofa. In this model, different input data from the wellness sofa is processed using a convolutional neural network (CNN) model, and a multimodal fusion technique is used to combine these output feature vectors. A unified representation of the older adult users’ real-time posture and real-time emotion will be used by the wellness HCI module to generate control of the different adaptive modules to produce appropriate adaptive feedback interactions.

The camera captures video signals, which are then preprocessed by resizing and grayscale conversion to enable face detection of older adult users. Using the OpenCV library, once the user’s face is detected by applying a pretrained machine learning model, the DeepFace model is used to extract facial features and identify the user’s emotional state. Using a CNN, the extracted facial features are used to identify the user’s emotion and determine sentiment tuning. Once the real-time emotional state of the older adult users is identified, the type of music required by the recreational sofa can be determined to suit the users’ changing emotions. Adjusting the music of the wellness sofa can be done using the emotional state adjustment determined in the previous step to adjust the music of the wellness sofa to suit the emotional state of the user. Finally, the process can be repeated many times to ensure that the Convalescent Sofa adapts to the real-time emotions of the older adult users in real time, as shown in Textbox 1.

Textbox 1. **Emotion recognition module framework.**

# Import necessary libraries

import cv2

import numpy as np

from deepface import DeepFace

# Capture the video feed

cap = cv2.VideoCapture(0)

……

  for (x,y,w,h) in faces:

   # Extract the facial features using pretrained machine learning models

  face_roi = frame[y:y+h, x:x+w]

  img = cv2.resize(face_roi, (224, 224))

  img = np.expand_dims(img, axis=0)

  img = img / 255.0

  embeddings = DeepFace.represent(img)

  # Recognize the user's emotion using pretrained machine learning models

  svm = cv2.ml.SVM_load('emotion_detection_svm.xml')

  pred = svm.predict(embeddings)[1][0][0]

  # Determine the emotion adjustment needed for the smart sofa

  if pred == 0:

   emotion_adjustment = 5

  elif pred == 1:

   emotion_adjustment = -5

### Materials

**Figure 2.**
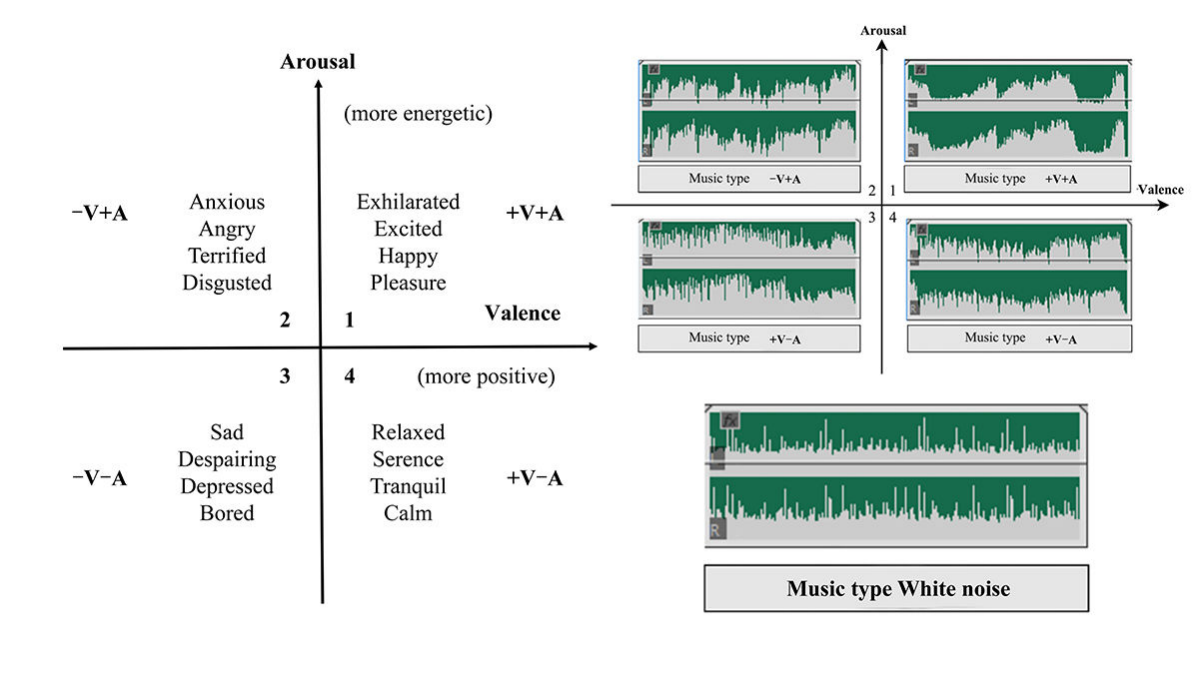
VA emotion models as well as experimental stimulus material. VA: Valence-Arousal.

The generic continuous dimension model that has been widely adopted in music emotion recognition research is the Circomplex model, also known as the VA model [48]. Research shows that specific musical characteristics are significantly correlated with Valence and Arousal [49]. For example, +V +A refers to a fast tempo, major key, and bright timbre. –V+ A refers to dissonant chords and a fast, irregular rhythm. –V –A refers to a slow tempo, minor key, and dull timbre. +V A refers to slow yet harmonious harmonies. Existing studies have demonstrated that bodily sensations and emotions triggered by music and based on the VA model show consistency across different cultures [50]. In this experiment, 4 pure music genres conforming to the 4 quadrants of the VA model were selected, and the stimulus materials were named A1-A4, that is, A1: +V +A, A2: –V+A, A3: –V–A, A4: +V A, as shown in Figure 2. A control group was set up using white noise to simulate white noise to simulate the daily life environment, and the stimulus material was named A0. The use of white noise provided a constant and semantically neutral auditory background, helping to avoid interference from background sounds on participants’ emotional responses [51].

### Participants

Sample size is critical to the reliability and representativeness of research findings. Following the standards proposed by Cohen [52], this study used G*Power software (Heinrich Heine University Düsseldorf, Germany) [53] to estimate the required sample size, selecting the within-factors repeated measures ANOVA design. The parameters included an alpha level of 0.05, a power level of 0.95, a medium effect size of 0.25, and a number of groups of 1. This setup yielded a total sample size requirement of 31 participants. In addition, to ensure greater statistical robustness and account for potential data loss among older adult participants during the experiment, the final sample size was further adjusted. This sample size is also consistent with previous EEG studies investigating music perception and stimulation in similar experimental contexts [54,55]. Therefore, a total of 68 older adult participants aged between 60 and 75 years were recruited for this experiment. All participants were recruited from various regions across China, with culturally diverse backgrounds and different living environments.

Among the participants, there were 34 female participants (n=34) with an average height of 160 cm and a mean BMI of 25.5, and 34 male participants (n=34) with an average height of 171 cm and a mean BMI of 24.86. All participants were required to be free of chronic diseases of the ear, back, and legs and in good physical condition.

### Ethical Considerations

This experiment was conducted in strict accordance with ethical guidelines. Prior to the start of the experiment, the experimental procedures were fully explained to all participants, ensuring that the experiment would not pose any risks to them, and written informed consent was obtained from all participants. Also, explicit consent has been granted by a participant to use their images for publication. The experiment has been reviewed and approved by the Ethics Committee of Nanjing Forestry University (Science and Technology Department of Nanjing Forestry University) with the approval number: 2023-03-29. To protect the privacy of participants, all collected data were anonymized before analysis, with no personal identifying information retained. Upon completion of the experiment, each participant received a compensation of 100 RMB (1 RMB= US $0.14) for their time and participation.

### Experimental Paradigm Design

The older adult participants were seated calmly in a comfortable chair. The experimenter prepared 5 preanalyzed audio clips, including 4 audio segments based on the VA model, as the stimulus material. Meanwhile, a white-noise environment was established as the control condition to ensure a consistent auditory ambience. The decibel level of these audio clips was 45 dB. This level was determined with reference to previous studies, which indicate that 45 dB falls within the range of low-noise and quiet environments for older adults, which helps maximize auditory clarity while preventing auditory fatigue or discomfort [56,57]. Although background noise levels in real smart home environments may exceed 45 dB, this value was chosen to obtain stable and reproducible ERP data in a controlled and quiet experimental setting, thereby minimizing the impact of external interference on older adult participants.

**Figure 3.**
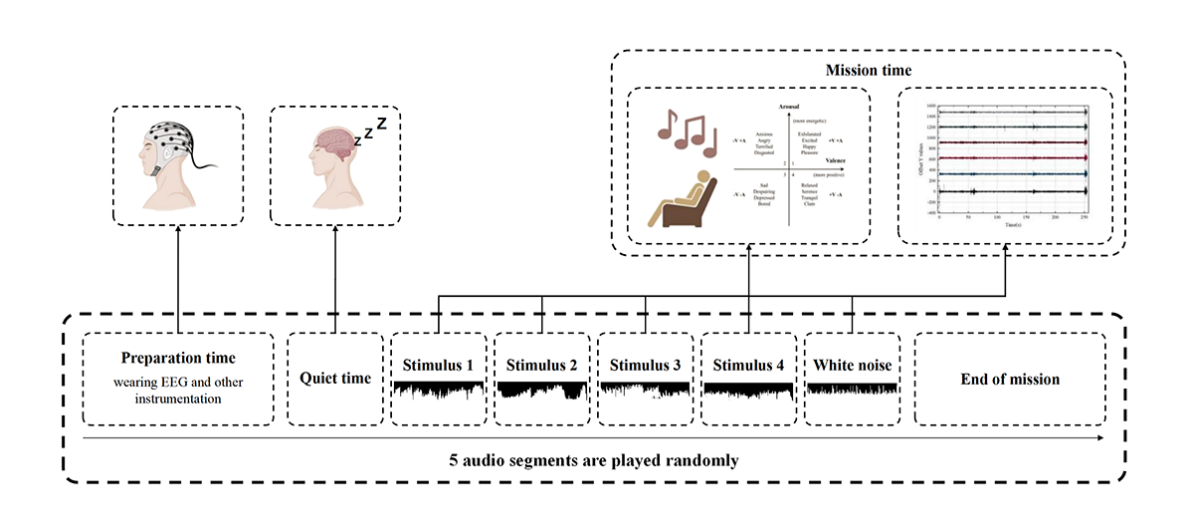
Experimental paradigm. The device worn by the participants was the Bitbrain hydroelectric electrode EEG, which had 16 channels, a rated voltage of 3.7 V, and a single-channel sampling rate of 256 Hz. The experiment started after the older adult participants entered the resting state. The experiment was divided into 5 recording stages, which were 5 sets of subexperiments, as a whole. In each stage, EEG signals were continuously recorded and saved separately, while each trial task was randomly ordered. EEG: electroencephalogram.

Prior to the start of the experiment, the experimental content and precautions were carefully explained to the participants. However, no information regarding the specific types of audio stimuli or their emotional attributes was disclosed to minimize expectation effects. Participants were also instructed to avoid vigorous movements or vocalization. After obtaining the participants’ consent, the informed consent form was signed, and preparations for the experiment, including scalp cleaning, began. The experimental equipment was worn by older adult participants throughout the experiment. The first formal recording was started after the participant indicated full understanding and initial control of the EEG signal. During the experiment, the system continuously monitored electrode connectivity. When head movement or electrode displacement caused channel dropouts or abnormal impedance, the acquisition interface issued a real-time alert. The experimenter immediately paused the task, readjusted the electrodes, and resumed recording once stable contact was restored, ensuring the consistency and comparability of EEG data across all recording stages. The EEG signals were recorded continuously in each session and saved separately. All trial tasks were presented in a randomized order to effectively minimize order and learning effects. The duration of the entire experiment was 15‐20 minutes, and the experimental paradigm is shown in Figure 3.

### EEG Recording and Preprocessing

**Figure 4.**
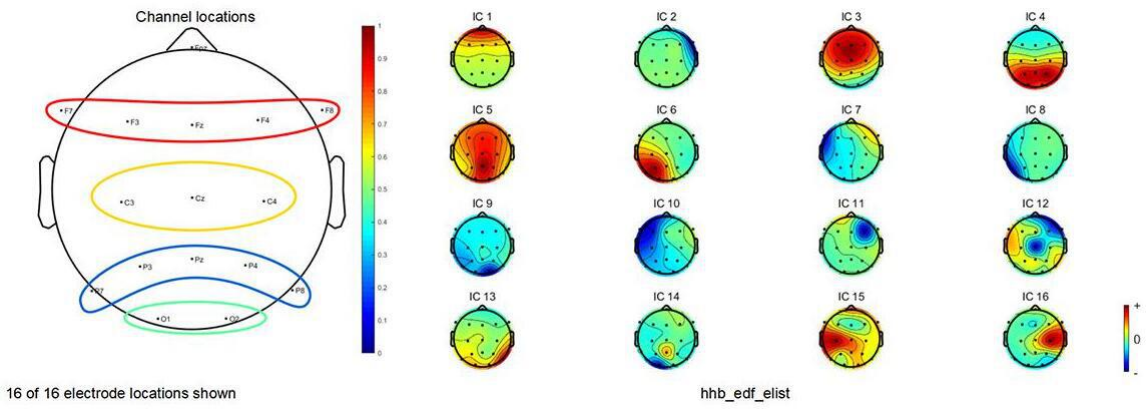
Diagram of the 16 electrode positions selected according to the international 10‐20. EEG electrodes in the 10‐20 system. The area within the red circle represents the frontal lobe (Fpz, **F7, F3, FZ, F4, and F8**), the yellow area represents the central region (**C3, CZ, and C4**), the blue area represents the parietal lobe (**P3, PZ, P4, P7, and P8**), and the green area represents the occipital lobe (**O1 and O2**). EEG: electroencephalogram.

The EEG signal was acquired according to the international 10‐20 system lead setup, with 16 electrodes selected, mainly placed in the frontal, parietal, and central regions, and covering the left and right hemispheres of the brain and the midline region, as shown in Figure 4. This study used a 16-channel EEG acquisition system, a configuration widely used in research on music-induced emotion and cognitive processing [58,59]. Although the number of electrodes was relatively limited, the layout covered key brain regions associated with emotional processing, auditory stimulation, and attentional allocation, effectively capturing the main activity characteristics of typical ERP components such as P200, P300, and N400 [60]. In addition, considering that the participants in this study were older adults, using fewer electrodes helped maintain signal quality while reducing physiological fatigue and discomfort caused by prolonged experiments, thereby improving experimental compliance and data stability. Based on the experimental acquisition parameters, it was estimated that approximately 65,313 data points could be collected per channel for each participant. After excluding 2 invalid samples, the total size of the raw EEG dataset in this study was 65,313×16×66, amounting to approximately 68,970,528 signal data points. This data volume resulted from the normal accumulation determined by the device sampling parameters and experimental design, without any redundant or oversampling processes; therefore, it did not introduce bias into the results.

During the acquisition process, the reference and ground electrodes were placed near the Cz electrode and the anterior part of the scalp, respectively. EEG signal preprocessing was conducted using MATLAB with the EEGLAB 2020 toolbox. The acquired .edf files were imported into EEGLAB 2020, and channel localization was performed based on the MNI Coordinate Systems standard brain imaging model. Sixteen channels were identified as Fpz, F7, F3, Fz, F4, F8, C3, Cz, C4, P7, P3, Pz, P4, P8, O1, and O2, as shown in Figure 4.

**Figure 5.**
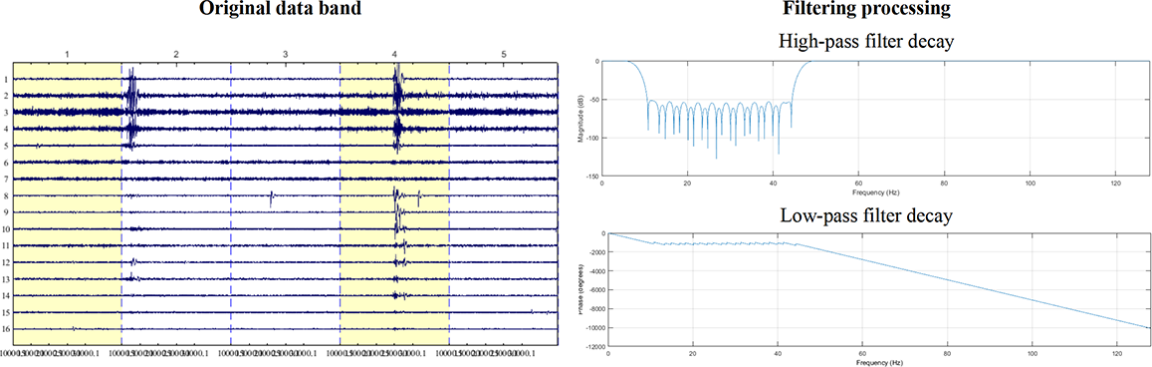
Comparison between the original band of the electroencephalogram and the data processed by filtering.

EEG data preprocessing was performed on the Jinfa EEG Signal Analysis Platform. First, all recordings were visually inspected by researchers to remove segments with obvious artifacts or discontinuities. A zero-phase finite impulse response filter was then applied for band-pass filtering (0.1‐30 Hz), and a notch filter was used to eliminate 50 Hz power-line noise. The original and filtered signals are shown in Figure 5. The data were subsequently rereferenced to the average and interpolated for any bad channels. Artifact removal was based on the standardized pipeline of Infomax independent component analysis and canonical correlation analysis built into the ErgoLAB Man-Machine-Environment Testing Cloud Platform (ErgoLAB 3.0). Identification of artifact components referenced conventional criteria commonly adopted in EEG research. Independent component analysis–derived components were identified and rejected based on their temporal characteristics, scalp topographies, and spectral features corresponding to ocular or muscular artifacts. To further improve signal quality, canonical correlation analysis was applied to remove residual artifacts. For the ERP analysis, the event-related time window was set from −200 to 800 milliseconds, with a baseline period of −200 milliseconds and a measurement window of 200 to 400 milliseconds.

### EEG Bands

The EEG bands in this experiment are divided into 5 bands. Gamma waves play an important role in the cognitive activity of the human brain and in higher-level activities such as the transmission of information in the brain, integrated processing, and feedback [61-63]. Beta waves, with amplitudes of 5-20 μV, are the fastest of the EEG waves and are more pronounced in the frontal and central regions [64]. Alpha waves, with amplitudes of 20-100 μV, are the fastest of the EEG waves and are more pronounced in the occipital and parietal regions [65]. Theta waves have an amplitude of 20 to 150 μV and are the slowest of the EEG waves, generally more pronounced in the parietal and temporal regions [66-69]. Delta waves, which typically exhibit amplitudes of up to approximately 200 μV, are the slowest type of EEG activity and are mainly observed in the frontal and occipital regions and generally occur during deep sleep, making them difficult to capture during the waking state [70-73]. The brain can be divided into parietal, temporal, frontal, and occipital lobes, of which the parietal lobe is mainly responsible for processing internal feedback, such as skeletal muscles and limbs [74-77].

### ERP Data

ERP is a high-level functional potential of the human brain [69]. ERPs are changes in brain potentials associated with cognitive processes such as judgment, attention, perception, decision-making, and working memory content [78]. Recent studies have shown that the amplitude and latency of ERPs are considered to be very important indicators of cognitive load in humans.

There are 3 key parameters of ERP, namely amplitude, latency, and distribution on the scalp [79]. By calculating the average amplitude of the ERP within the corresponding time window, the differences between different conditions can be analyzed. The latency represents the time interval from the onset of the stimulus to the occurrence of the ERP peak, usually expressed in milliseconds. The distribution of the ERP on the scalp can reveal which areas of the brain are functioning when the stimulus appears. The ERP with these 3 key parameters can reveal individuals’ cognitive and emotional processing under specific conditions [80,81].

ERPs are typically defined as time-locked neural responses to brief stimuli. Nevertheless, previous studies have shown that ERP components with typical temporal characteristics can also be observed under continuous or natural musical stimulation, particularly when different audio segments exhibit distinct conditional variations [82-84]. In this experiment, each music clip was treated as an independent event type. The onset of each music clip was set as the event trigger point (time=0 milliseconds), and a time window of −200 to 2000 milliseconds was extracted with a 200 milliseconds baseline correction applied. For each participant, the trial waveforms of the same music type were extracted and averaged to obtain the ERP, which reflects the cognitive and emotional processing of different music types in older adults.

## Results

### EEG Band Analysis

This experiment mainly collected the EEG changes of the older adult participants in the leisure state, so the electrode position of the parietal lobe was mainly selected as the key observation object, and these 3 bands of θ, α, and β were selected as the main discriminating parameters of the brain fatigue in this experiment. The power of the EEG bands under the stimulation materials of A0-A4 is shown in Table 1.

**Table 1.** Electroencephalogram band power under A0-A4 stimulation material.

Stimulation materials and project	δ	θ	α	β	γ
A0					
Total power	−4.89	16.43	15.18	13.38	8.29
Average power	−9.66	10.41	7.40	1.34	−4.50
Percentage power (%)	0.31	41.54	31.18	20.6	6.38
A1					
Total power	−25.49	10.18	12.27	12.64	9.31
Average power	−30.27	4.16	4.49	0.60	−3.48
Percentage power (%)	0.01	19.22	31.14	33.89	15.74
A2					
Total power	−2.08	20.35	19.51	16.26	9.28
Average power	−6.85	14.33	11.72	4.22	−3.51
Percentage power (%)	0.25	43.56	35.82	16.97	3.4
A3					
Total power	−18.42	12.18	13.27	12.71	7.91
Average power	−23.19	6.16	5.49	0.67	−4.88
Percentage power (%)	0.02	26.36	33.91	29.83	9.87
A4					
Total power	−18.39	13.29	15.29	12.37	6.15
Average power	−23.16	7.26	7.51	0.33	−6.64
Percentage power (%)	0.02	27.84	44.20	22.56	5.38

The total power value of the theta band was 20. Thirty-five at the highest in stimulus material A2 and occupied 43.56% of the power in this stimulus band, which can be monitored in the sleepy state, especially in the depressed or frustrated state. This indirectly verifies that the stimulus material A2, that is, –V+ A, can induce negative emotions such as sleepiness and frustration. The total power value of the alpha band accounted for the highest percentage of power, which was 44.20% when using stimulus material A4. Clinical research has shown that this is related to spatial attention. Therefore, the stimulus material A4 (ie,+ V A) may facilitate higher levels of creative thinking in older adult participants during cognitive tasks [85]. For stimulus material A1, the beta band’s total power value accounted for the highest percentage, 33.89%. Clinically, this band is linked to emotional arousal, indicating that A1 (+ V +A) can easily trigger emotional fluctuations in older adult participants.

**Figure 6.**
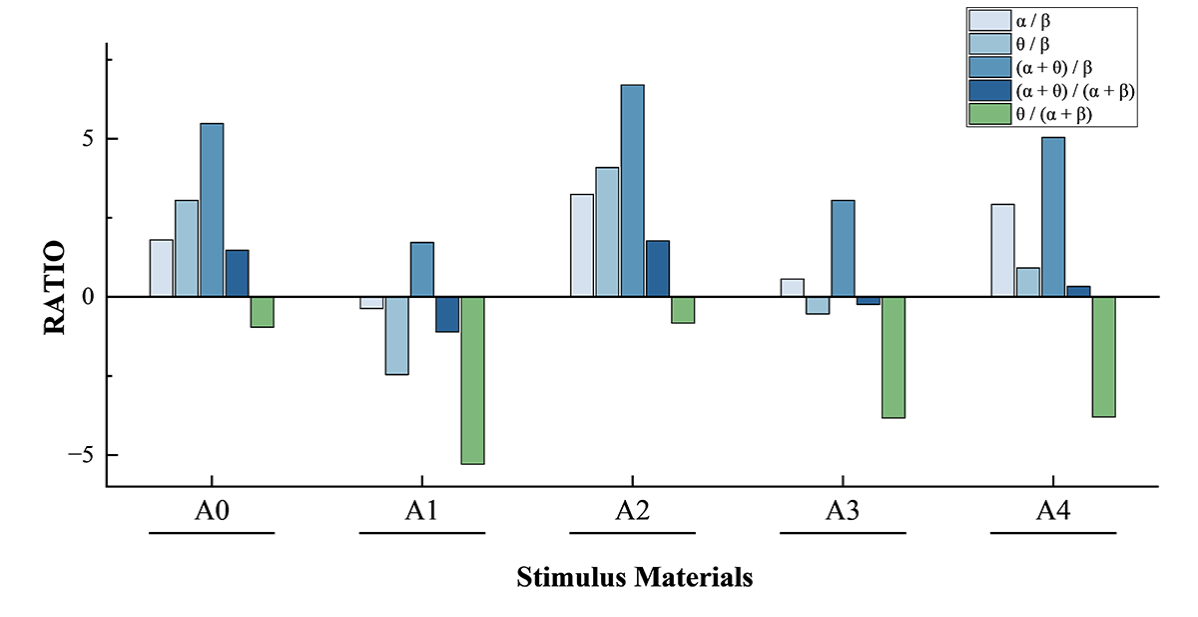
Brain wave band ratio under A0-A4 stimulation material state.

The ratio of theta to beta wave energy, F(θ/ β), and the ratio of the sum of theta and alpha wave energy to beta wave energy, F(α + θ) / β, are used as characteristic quantities for EEG fatigue assessment [47,48]. As F(θ / β) rises, the degree of fatigue further increases. The EEG band ratios for the A0 to A4 stimulus material states were derived by further processing of the ratios in Table 1, as shown in Figure 6.

This study found that different musical stimuli have an impact on the mental workload of older adult participants to some extent. The F(α + θ) / β value was much lower than the F(α + θ) / β values for the other 4 musical stimulus states when the stimulus material was A1. So the EEG fatigue level of the older adult participants was lowest when the stimulus material was A1, that is, +V+A, and highest when the stimulus material was A2, that is, –V+A. The F (α + θ) / β value of stimulus material A3 indicates that it can be selected as an auxiliary stimulus in moderate situations. The effect of music stimulation on the brain load of the older adult participants was therefore reflected in some way. It can be seen that music stimulates the brain waves of older adult participants and that different types of music can be used in daily life to induce emotions in older adult participants. +V+A music, such as exhilaration, excitement, and happiness, helps to reduce fatigue, while music of the −V+A type (eg, evoking anxiety, anger, or panic) induces negative emotions and increases brain load [86].

### ERP Data Analysis

The frontal zone electrodes (F7, F8, FPZ, and FZ), central zone electrodes (CZ), parietal zone electrodes (P3, P4, P7, P8, and Pz), and occipital zone electrodes (O1 and O2) were selected for the analysis. EEG data from each condition were analyzed using Statistical Product Service Solutions (SPSS; IBM Corp) software. Given that the objective of this study was to explore differences in EEG activity among older adult participants under various music stimulation conditions, one-way ANOVA with least significant difference (LSD) post hoc comparisons was used to maintain sensitivity in detecting potential effects during this exploratory phase. The regions (left, middle, and right) were divided for the evoked P200, P300, and N400 EEG components, as shown in Table 2. The selection of P200, P300, and N400 components as analytical indicators was based on their respective neurophysiological significance in emotional and cognitive processing. The P200 component typically occurs approximately 200 milliseconds after stimulus onset and can be regarded as an initial perceptual response to auditory stimulation [87]. The P300 component usually emerges around 300 milliseconds after stimulus onset, reflecting neural activity associated with attention allocation, working memory, and cognitive evaluation processes [88]. The N400 component generally appears approximately 400 milliseconds after stimulus onset and represents a neural response to semantic incongruity or expectancy violation [89]. By analyzing these 3 components, individual emotional responses can be comprehensively evaluated across different stages of cognitive processing.

**Table 2.** Division of evoked potentials into brain regions.

EEG^a^ component	Left area	Middle area	Right area
P200	P7, P3, O1	PZ	P4, P8, O2
P300	F7	FPZ, FZ	F8
N400	O1	CZ	O2

a EEG: electroencephalogram.

### Statistical Analysis of P200 Components

For the P200 EEG component, the latency averages of each electrode (P7, P3, O1, PZ, P4, P8, and O2) were obtained by total superimposed averaging of the participants’ waveforms in a total of 5 stimulus material states from A0 to A4, as shown in Table 3.

**Table 3.** A0-A4 mean latency for each electrode of the P200 component in the stimulated.

Stimulation materials	P7	P3	O1	PZ	P4	P8	O2
A0	0.094	0.086	0.095	0.088	0.093	0.091	0.088
A1	0.189	0.067	0.165	0.087	0.184	0.086	0.154
A2	0.034	−0.147	−0.099	−0.470	0.027	0.303	0.108
A3	0.072	0.003	0.050	0.080	0.181	0.079	0.110
A4	0.053	0.062	0.680	0.069	0.072	0.060	0.074

**Figure 7.**
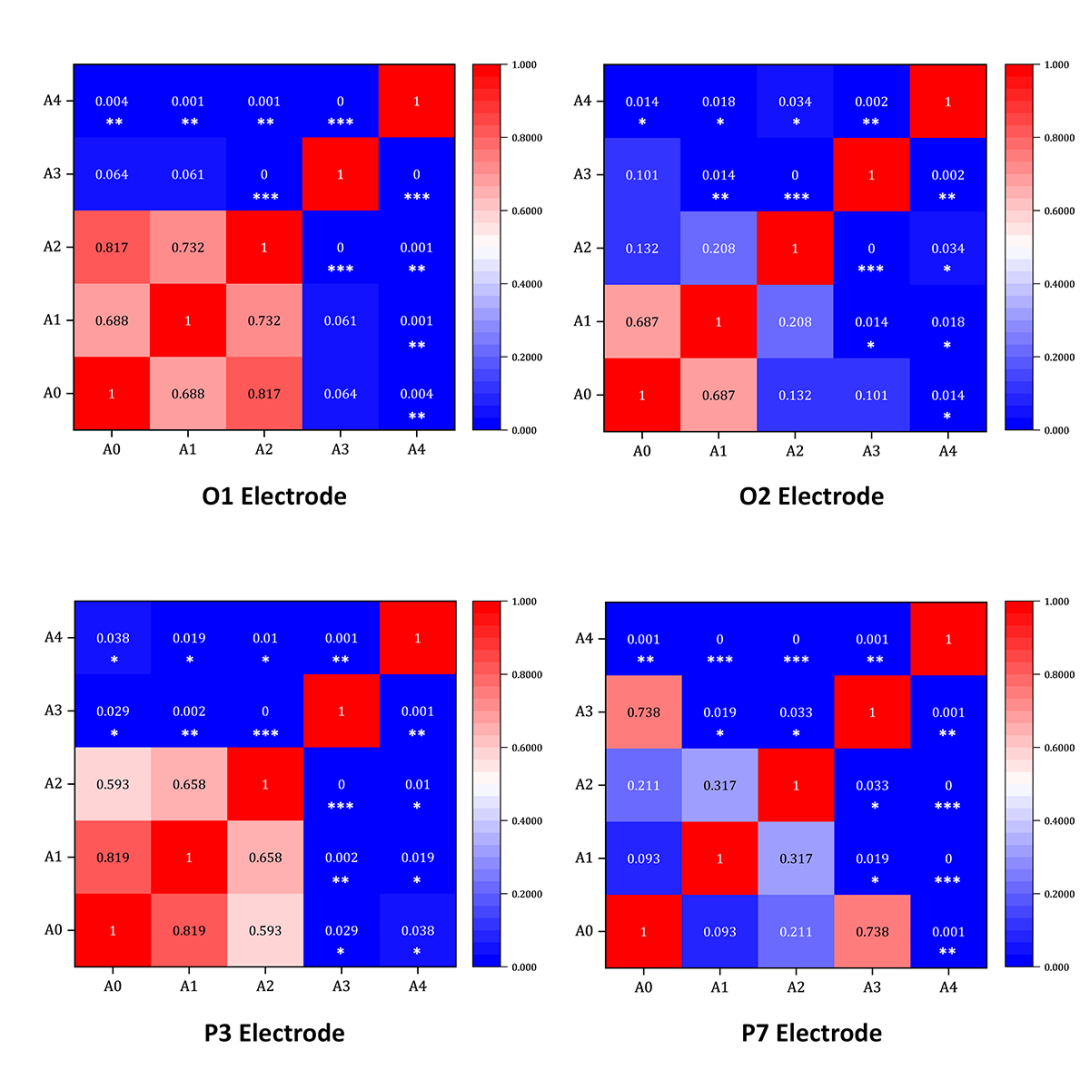
Analysis of the P200 heat map under the states of stimulus materials A0-A4. Each cell in the heatmap represents the *P* value of the pairwise comparison between conditions, with deeper colors indicating more significant differences. The diagonal cells represent self-comparisons and are displayed as 1. The numerical values in the figure correspond to the *P* values from the least significant difference post hoc test, and significance levels are denoted as *P<.05, **P<.01, and ***P<.001.

Analysis of the mean values of the latency of each electrode for the P200 component in the A0-A4 stimulation material condition shows that there were differences in the mean values of the latency of each electrode for the P200 component. In the A0 stimulation condition, the mean value of the O1 channel was the largest, and the mean value of the P3 channel was the smallest; in the A1 stimulation condition, the mean value of the P7 channel was the largest, and the mean value of the P3 channel was the smallest. In the A2 stimulation condition, the mean value of the P8 channel was the largest, and the mean value of the PZ channel was the smallest. In the A3 stimulation condition, the mean value of the P4 channel was the largest, and the mean value of the P3 channel was the smallest. In the A4 stimulation condition, the mean value of the O2 channel was the largest, and the mean value of the P7 channel was the smallest. The P200 component data obtained were imported into the SPSS mathematical analysis software. One-way ANOVA was used to test the influence of different stimulus materials on the P200 components of each electrode, and the means and SDs were calculated, as shown in Table 4. To further analyze the differences, we used the LSD method for post hoc multiple comparisons, as depicted in Figure 7.

**Table 4.** P200 component ANOVA results.

Variable and value	Mean (SD)	*F*_1_-score	*P* value
P7		12.342	<.001
A0	2.382 (8.783)		
A1	−0.276 (6.735)		
A2	0.750 (2.604)		
A3	2.903 (6.550)		
A4	18.507 (32.256)		
Total	4.853 (16.933)		
P3		6.243	<.001
A0	1.638 (9.222)		
A1	1.290 (5.440)		
A2	0.914 (2.482)		
A3	−1.369 (2.579)		
A4	7.605 (17.856)		
Total	2.016 (9.838)		
O1		9.931	<.001
A0	1.002 (7.944)		
A1	0.450 (5.555)		
A2	0.734 (1.840)		
A3	−1.279 (3.282)		
A4	8.307 (15.511)		
Total	1.843 (8.930)		
PZ		7.216	<.001
A0	0.552 (8.025)		
A1	1.358 (3.445)		
A2	1.009 (1.253)		
A3	−2.218 (2.567)		
A4	5.871 (14.469)		
Total	1.314 (8.039)		
P4		10.441	<.001
A0	1.536 (6.285)		
A1	0.954 (3.973)		
A2	1.262 (1.878)		
A3	1.262 (3.524)		
A4	7.185 (14.409)		
Total	1.734 (8.014)		
P8		1.683	.155
A0	0.330 (0.492)		
A1	0.598 (4.628)		
A2	1.224 (1.939)		
A3	−1.699 (3.888)		
A4	2.007 (14.895)		
Total	0.492 (7.590)		
O2		6.408	<.001
A0	−0.126 (5.572)		
A1	0.280 (4.399)		
A2	1.092 (1.087)		
A3	−1.623 (3.135)		
A4	6.474 (17.694)		
Total	6.474 (9.023)		

Further analysis of the potential amplitudes of the P200 component revealed that there were significant differences in different types of musical stimulus materials on the electrodes P7, P3, O1, and O2. For the variable P7 (*F*_4, 268_=12.342, *P*<.001, η²=0.168), the LSD test showed that the amplitude of the A1 stimulation (mean −0.276) was significantly lower than that of the A4 stimulation (mean 18.507, *P*<.001). For the variable P3 (*F*_4, 268_=6.243, *P*<.001, η²=0.092), the LSD test showed that the amplitude of the A2 stimulation (mean 0.914) was significantly lower than that of the A4 stimulation (mean 7.605, *P*=.010). For the variable O1 (*F*_4, 268_=9.931, *P*<.001, η²=0.140), the LSD test showed that the amplitude of the A1 stimulation (mean 0.450) was significantly lower than that of the A4 stimulation (mean 8.307, *P*=.001). For the variable O2 (*F*_4, 268_=6.408, *P*<.001, η²=0.095), the LSD test showed that the amplitude of the A0 stimulation (mean −0.126) was significantly lower than that of the A4 stimulation (mean 6.474, *P*=.014).

A detailed analysis of the heatmap shown in Figure 7 revealed that A4 stimulation, that is, +V A, had the strongest activation, with significantly higher average potential amplitudes across all channels compared with other stimuli and the A0 control. Musical stimuli also differentially activated the left parietal and occipital lobes.

### Statistical Analysis of P300 Components

For the P300 EEG component, the voltage-phase EEG data of each electrode in the period of 150‐250 milliseconds were extracted. The latency averages of each electrode of P300 (F7, FPZ, FZ, and F8) were obtained by averaging the superimpositions of the participants’ waveforms in 5 stimulus material states of A0-A4, as shown in Table 5.

**Table 5.** A0-A4 mean latency for each electrode of the P300 component in the stimulated.

Stimulation materials	F7	FPZ	FZ	F8
A0	0.037	0.078	0.073	0.112
A1	−0.015	−0.407	0.071	−0.010
A2	−0.527	−0.470	0.274	0.303
A3	−0.340	−0.336	0.141	0.189
A4	0.046	0.167	0.126	0.109

**Figure 8.**
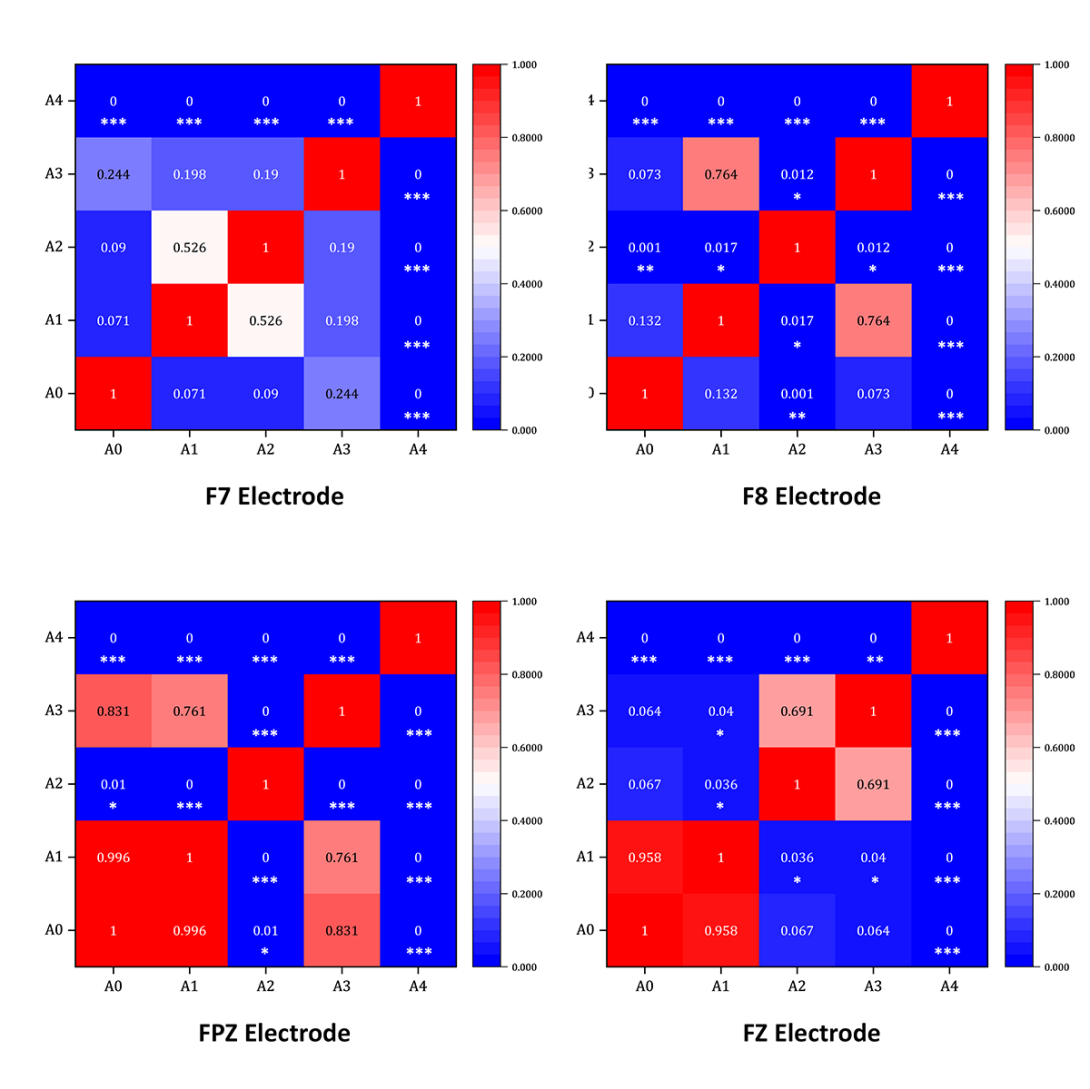
Analysis of the heat map of P300 at each electrode under the states of stimulus material A0-A1. Each cell in the heatmap represents the *P* value of the pairwise comparison between conditions, with deeper colors indicating more significant differences. The diagonal cells represent self-comparisons and are displayed as 1. The numerical values in the figure correspond to the *P* values from the least significant difference post hoc test, and significance levels are denoted as *P<.05, **P<.01, and ***P<.001.

Analysis of the latency averages for each electrode of the P300 component in the A0-A4 stimulus material condition shows that there were differences in the latency averages for each electrode of the P300 component. Under the A0 stimulation condition, the mean value of the F8 channel was the largest, and the mean value of the F7 channel was the smallest. Under the A1 stimulation condition, the mean value of the FZ channel was the largest, and the mean value of the FPZ channel was the smallest. Under the A2 stimulation condition, the mean value of the F8 channel was the largest, and the mean value of the F7 channel was the smallest. Under the A3 stimulation condition, the mean value of the F8 channel was the largest, and the mean value of the F7 channel was the smallest. Under the A4 stimulation conditions, the mean value of the FPZ channels was the largest, and the mean value of the F7 channels was the smallest. The obtained data of the P300 component were calculated for the means and SDs and subjected to one-way ANOVA, as shown in Table 6. The LSD method was used for post hoc multiple comparisons to further analyze the differences, as illustrated in Figure 8.

**Table 6.** P300 component ANOVA results.

Variable and value	Mean (SD)	*F*_1_-score	*P* value
F7		17.065	<.001
A0	1.608 (6.967)		
A1	−0.482 (4.099)		
A2	−0.101 (1.062)		
A3	0.387 (2.386)		
A4	13.404 (20.879)		
Total	2.963 (11.307)		
FPZ		668.888	<.001
A0	1.044 (4.771)		
A1	−0.152 (2.882)		
A2	−1.249 (1.351)		
A3	−0.307 (2.232)		
A4	44.444 (28.429)		
Total	8.756 (22.062)		
FZ		30.449	<.001
A0	−1.152 (7.616)		
A1	−1.146 (4.626)		
A2	−4.109 (2.355)		
A3	−0.900 (3.342)		
A4	57.083 (12.804)		
Total	9.955 (24.699)		
F8		117.799	<.001
A0	0.990 (7.455)		
A1	0.918 (6.245)		
A2	−0.986 (1.086)		
A3	−1.197 (3.580)		
A4	12.585 (12.766)		
Total	2.462 (8.945)		

A further analysis of the potential amplitudes of the P300 component revealed that there were significant differences and activation phenomena in the prefrontal lobe region for the musical stimulation materials. Specifically, for the variable F7 (*F*_4, 268_=17.065, *P*<.001, η²=0.218), the LSD test showed that the average potential amplitude under the A2 stimulation material (mean −0.101) was much lower than that under the A4 stimulation material (mean 13.404, *P*<.001). For the variable FPZ (*F*_4, 268_=668.888, *P*<.001, η²=0.916), the LSD test showed that the average potential amplitude under the A1 stimulation material (mean −1.146) was much lower than that under the A4 stimulation material (mean 57.083, *P*<.001). For the variable FZ (*F*_4, 268_=30.449, *P*<.001, η²=0.332), the LSD test showed that the average potential amplitude under the A3 stimulation material (mean −1.197) was much lower than that under the A4 stimulation material (mean 12.585, *P*<.001). For the variable F8 (*F*_4, 268_=117.799, *P*<.001, η²=0.658), the LSD test showed that the average potential amplitude under the A1 stimulation material (mean −0.152) was much lower than that under the A4 stimulation material (mean 44.444, *P*<.001).

A detailed analysis of the heatmap shown in Figure 8 reveals that there are significant differences among the different types of musical stimulation materials at the F7, FPZ, FZ, and F8 electrodes. The average potential amplitudes of the variables for each channel under the A4 stimulation material, that is, +V A, were much higher than those under the other 3 types of stimulation materials and the A0 stimulation material of the control group.

### Statistical Analysis of N400 Components

For the N400 EEG components, the voltage EEG data of each electrode in the period of 150‐250 milliseconds were extracted. By averaging the total superposition of the participants’ waveforms in the 5 stimulus material states from A0-A4, the latency averages of each electrode of the N400 (O1, CZ, and O2) were obtained, as shown in Table 7.

**Table 7.** A0-A4 mean latency for each electrode of the N400 component in the stimulated.

Stimulation materials	O1	CZ	O2
A0	0.095	0.088	0.072
A1	0.165	0.120	0.154
A2	−0.099	0.116	0.108
A3	0.050	0.136	0.110
A4	0.069	0.076	0.074

**Figure 9.**
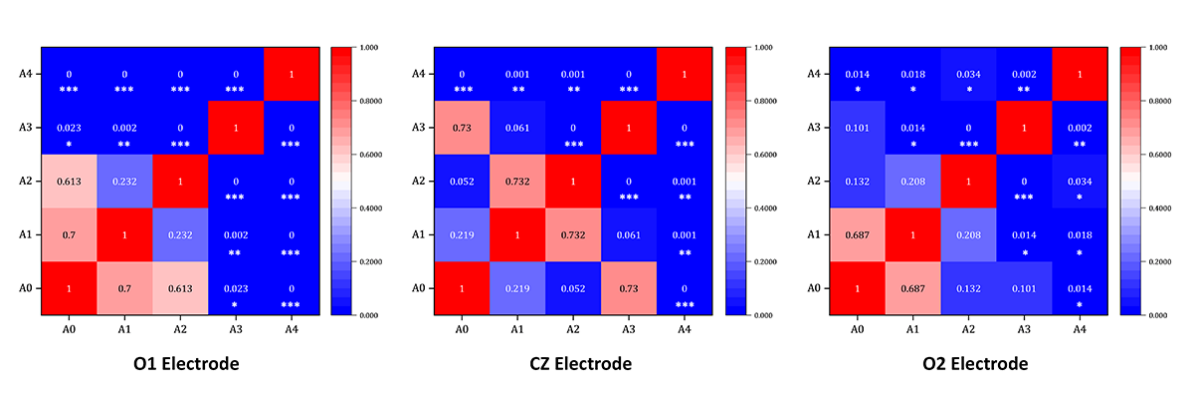
Analysis of the heat map of N400 at each electrode under the states of stimulus material A0-A1. Each cell in the heatmap represents the *P* value of the pairwise comparison between conditions, with deeper colors indicating more significant differences. The diagonal cells represent self-comparisons and are displayed as 1. The numerical values in the figure correspond to the *P* values from the least significant difference post hoc test, and significance levels are denoted as *P<.05, **P<.01, and ***P<.001.

Analysis of the mean latency values of each electrode of the N400 component under the stimulation material conditions of A0-A4 shows that there are differences in the mean latency values of each electrode of the N400 component. Under the A0 stimulation condition, the mean value of the O1 channel was the largest, and the mean value of the O2 channel was the smallest. Under the A1 stimulation condition, the mean value of the O1 channel was the largest, and the mean value of the CZ channel was the smallest. Under the A2 stimulation condition, the mean value of the CZ channel was the largest, and the mean value of the O1 channel was the smallest. Under the A3 stimulation condition, the mean value of the CZ channel was the largest, and the mean value of the O1 channel was the smallest. Under the A4 stimulation condition, the mean value of the CZ channel was the largest, and the mean value of the O1 channel was the smallest. The obtained data of the P300 component were calculated for the means and SDs and subjected to one-way ANOVA, as shown in Table 8. The LSD method was used for post hoc multiple comparisons to further analyze the differences, as illustrated in Figure 9.

**Table 8.** N400 component ANOVA results.

Variable and value	Mean (SD)	*F*_1_-score	*P* value
O1		63.016	<.001
A0	1.002 (7.944)		
A1	1.568 (6.642)		
A2	0.430 (0.808)		
A3	−1.806 (3.316		
A4	21.713 (15.859)		
Total	4.581 (12.160)		
CZ		12.095	<.001
A0	−0.954 (5.789)		
A1	0.450 (5.555)		
A2	0.734 (1.840)		
A3	−1.279 (3.282)		
A4	8.307 (15.511)		
Total	1.452 (8.672)		
O2		6.408	<.001
A0	−0.126 (5.572)		
A1	0.280 (4.399)		
A2	1.092 (1.087)		
A3	−1.623 (3.135)		
A4	6.474 (17.694)		
Total	1.219 (9.023)		

A further analysis of the potential amplitudes of the N400 component reveals that there are obvious differences and degrees of activation in the occipital lobe region and the central line region of the temporal area caused by the musical stimulation materials. The analysis shows that for the variable O1 (*F*_4, 268_=63.016, *P*<.001, η²=0.507), the LSD test reveals that the average potential amplitude under the A2 stimulation material (mean 0.430) is much lower than that under the A4 stimulation material (mean 21.713, *P*<.001). For the variable CZ (*F*_4, 268_=12.095, *P*<.001, η²=0.165), the LSD test indicates that the average potential amplitude under the A1 stimulation material (mean 0.450) is much lower than that under the A4 stimulation material (mean 8.307, *P*<.001). For the variable O2 (*F*_4, 268_=6.408, *P*<.001, η²=0.095), the LSD test shows that the average potential amplitude under the A0 stimulation material (mean −0.126) is much lower than that under the A4 stimulation material (mean 6.474, *P*<.001).

A detailed analysis of the heatmap shown in Figure 9 reveals that there are significant differences among different types of musical stimulation materials at the O1, CZ, and O2 electrodes. In the N400 component, the average potential amplitudes of the variables for each channel under the A4 stimulation material, that is, +V A, are much higher than those under the other 3 types of stimulation materials and the A0 stimulation material of the control group.

## Discussion

### Principal Results

This study aims to establish a mapping relationship between musical parameters and emotional states based on a music emotion model, and to provide empirical evidence for the design of a music-interaction module for intelligent leisure sofas in home environments. Based on the VA model, 4 sets of musical stimulus materials were formulated, and the state responses of older adult participants while listening to different musical stimulus materials were collected by the Bitbrain hydropolar EEG imaging system. The experimental results indicated that different types of musical stimuli exerted significant effects on the EEG activity of older adults.

First, compared with the white noise control condition, all 4 types of musical stimuli exhibited significant differences in EEG activation levels, indicating that music stimulation can effectively influence the neural workload of older adult participants. This finding is consistent with previous research on the regulatory effects of music on neural activity. For instance, Kučikienė et al [90] reported that preferred music enhanced EEG power across multiple frequency bands, particularly within the α/β bands. Similarly, Yang et al [91] found significant associations between brain activity in different frequency bands and musical rhythm. In the present study, we observed that +V+A pleasurable, excited, and happy types of music elicited higher levels of EEG activation, which may be associated with increased α and β power and decreased θ activity, changes that facilitate positive emotional experiences and reduce fatigue [92]. In contrast, –V+ A anxious, angry, and extremely panicky types of music were characterized by higher proportions of θ activity and peak (α+θ)/β ratios, which may correspond to EEG mechanisms commonly observed during negative emotional stimulation, such as enhanced θ and suppressed α power [93].

Analysis of the ERP data revealed significant differences among the 4 types of musical stimuli for the P200, P300, and N400 components, reflecting the modulatory effects of music on cognitive processing and emotional responses in older adult participants. Regarding the P200 component, our study showed significant differences across electrode sites P7, P3, O1, and O2, primarily localized in the left parietal and occipital regions. This suggests that music can activate the brain’s perceptual and attentional networks at an early processing stage, facilitating the initial encoding of auditory information. Similarly, Patel et al [94] reported that musical stimuli could evoke early-stage neural responses for attention and perceptual processing, with the P200 component being closely linked to perceptual and selective attention processes. In addition, Portnova et al [95] found that the perception of emotional stimuli in comatose patients was associated with early P200 responses, whereas healthy individuals exhibited more complex ERP patterns.

Regarding the P300 component, its amplitude is closely associated with attention, working memory, and emotional states [96]. Evidence suggests that musical stimuli can modulate brain activity during later stages of cognitive processing, significantly influencing P300 amplitude. For instance, Pawlowski et al [97] noted that the P300 component is strongly related to cognitive evaluation and decision-making processes. In the present study, significant differences in P300 amplitudes were observed across electrode sites O1, Cz, and O2, with particularly prominent differences in activation in the occipital and temporal regions. These findings indicate that musical stimuli of different emotional types can significantly affect the allocation of attention and the level of cognitive evaluation in older adult participants, reflecting the specific modulatory role of music in brain cognitive processing.

Regarding the N400 component, although N400 was originally studied in the context of language processing, recent research has extended its application to the field of music to investigate the neural mechanisms underlying musical semantic processing. For example, Calma-Roddin et al [98] found that disharmonious or unexpected changes in familiar melodies elicited an N400-like effect similar to that observed in language, suggesting that music processing also involves semantic-related cognitive mechanisms. In our analysis of the N400 component, significant differences were observed across electrode sites F7, Fpz, Fz, and F8, particularly in the frontal region. This finding is consistent with previous research, indicating that musical stimuli can evoke brain neural responses associated with semantic processing and emotional evaluation. For instance, Portnova et al [95] reported that the N400 component is closely related to emotional perception and semantic processing, and that higher N400 amplitudes have been linked to the perception of emotional expression in sounds.

In summary, different types of musical stimuli exhibit significant differences in the brain activity of older adult participants. These results provide empirical evidence for the neural mechanisms underlying the regulatory effects of music on the emotions and cognition of older adults and offer a basis for the design of music-interaction regulation modules in smart home systems. From a theoretical perspective, the findings of this study further enrich the research framework regarding emotion-driven adaptability of home environments in smart home design [99] and support the necessity of introducing emotional response mechanisms in home environments [100]. Meanwhile, this study also provides empirical supplementation to the affective-rational theory [101,102]. The experimental results indicate an interaction between emotional experience and rational processing in the older adult users under musical stimulation. On this basis, the proposed music feedback mechanism can achieve adaptive regulation of users’ emotions by identifying the corresponding relationship between emotions and music, which aligns with the laws of human cognitive processing and people-oriented HCI models [103]. Finally, through a comprehensive analysis of musical stimuli and EEG responses, this study reveals the emotional interaction relationships of the older adult users in the context of smart homes. As Desmet et al [104] noted, emotional artifacts promote well-being and psychological balance by evoking positive emotional experiences. This provides an actionable theoretical basis and experimental support for the future design of emotional products for older adult users and the development of health-promoting interaction systems.

### Practice

The emotion recognition module plays a key role in the multimodal HCI system of the Recreation Sofa, which provides an immersive and personalized musical experience for the user. The module is designed to recognize the current behavioral and emotional state of the older adult user and adjust the music and interaction accordingly according to predefined criteria in the recreation module. This involves the integration of multiple sensors and algorithms to detect the emotional, behavioral, and activity levels of the older adult user and select music that matches their preferences and needs. For example, if an older user is feeling stressed or tired, the emotion recognition module can select calming or relaxing music to help them unwind. Or, if the older user is feeling energetic or happy, the music module can select more upbeat or lively music to enhance their emotions. If the older user is in a relaxed state, the emotion recognition module will select calm and soothing music to match the user’s emotional and behavioral state. The emotion recognition module also adjusts the volume and tempo of the music to the user’s preferences, ensuring a comfortable and enjoyable musical experience when using the lounge sofa. The emotion recognition module thus plays an important role in providing a personalized and adaptive user experience that is tailored to the user’s emotional and behavioral state. The emotion recognition module uses the predefined music matching criteria set in the Recreation module to select the appropriate type of music.

**Figure 10.**
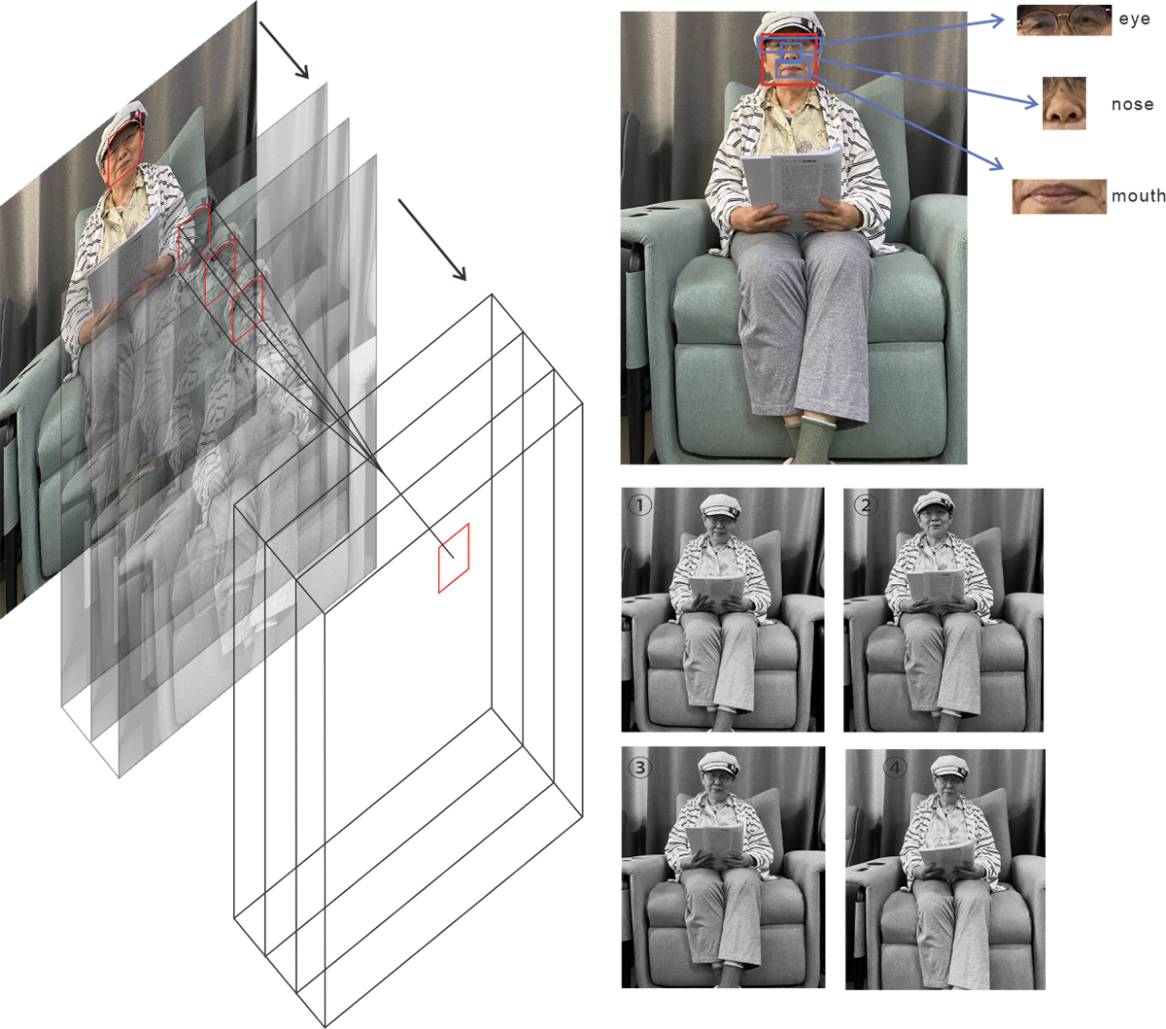
Model foundation framework.

The emotion recognition module carries out facial recognition of the older adult users through the Intel RealSense camera. The facial features are divided through the emotion recognition module, which mainly identifies the 3 main parts of the older adult users’ face, the eyes, nose, and corners of the mouth, and the recognition process is shown in Figure 10. During the recognition process, the captured image will be grayed out to determine the gender of the target user, establish where the target facial range of the user lies, extract the 3 main facial feature parts of the target user in turn, and determine the actual emotional state of the user through a comprehensive evaluation of the algorithm. At this stage, the module has been developed as a prototype to test the feasibility of facial emotion recognition and its integration effect in the interactive music system. The study adopts the CNN-DeepFace framework to process the facial images of 68 older adult participants in the EEG-music experiment. These images serve as the initial dataset for verifying the effectiveness of the emotion mapping and adaptive playback process. The purpose of this stage is not to train a new deep learning model, but to evaluate the technical feasibility and response accuracy of the system in a controlled environment.

The emotion recognition and music interaction module developed based on the conclusions of this experimental study aims not only to enhance user experience through its technical implementation, but also needs to be examined within the broader context of national, cultural, and ethical frameworks [105]. At the societal level, this study responds to the global trends of intelligent older adults’ care and home-based health promotion [106]; it seeks to improve the psychological and emotional well-being of the older adults in home environments through noninvasive emotion perception and matched music interventions. At the cultural level, the randomized controlled experiment conducted using EEG in this study prioritizes cultural universality in music selection and intervention strategies. Music based on the VA model was selected to avoid emotional biases or culturally insensitive interventions caused by cultural differences, thereby ensuring the cross-cultural acceptability of the experiment [107].

At the level of technical ethics and safety practice, the design integrating the interaction module with the leisure sofa adheres to the design guidelines for HCI safety [108]. The main hardware involved includes a speech recognition microphone array, an emotion recognition camera, and an intelligent audio playback device. At the communication level, the system primarily relies on wireless communication protocols such as Wi-Fi, Bluetooth, and LoRa, while realizing multimodal data interaction and scenario linkage through a smart home gateway. However, subsequent research and practice need to consider data security, a critical consideration in the smart home ecosystem, which requires balancing the protection of user privacy with the migration and linkage between smart products [109]. From the perspective of ethics and social well-being, this system aims to provide older adults with an intelligent companionship solution that offers emotional comfort and is oriented toward social care. It enables music to serve not only as feedback for product functions but also as a carrier for emotional care, which aligns with the goal of technology facilitating a better quality of life [110]. Meanwhile, the system should also emphasize that technology should be people-oriented, explainable, and supportive of social dialogue. This requires prioritizing design values over technical implementation, rather than merely pursuing performance optimization [111].

### Comparison With Prior Work

The analogies between the findings of this paper and the existing literature related to music emotion recognition or regulation are shown in Table 9.

**Table 9.** Correlation of the findings of this paper with the existing literature on musical emotion recognition and regulation.

Comparison	This work	Yang et al [112]	Takashima et al [113]	Wang et al [114]	Sutcliffe et al [115]	Vieillard and Gilet [116]
Research participants	Older adults	General	Datasets	Youth and older adults	Youth and older adults	Youth and older adults
Research methods	Model building, experiments, physiological data analysis	Model building, performance testing	Model building, dataset experiments	Experiments, physiological data analysis	Experiments, physiological data analysis	Experiments, physiological data analysis
Research tools	EEG^a^ and ERP^b^	Multimodal research	EMER-CL^c^	Residual convolutional network	Research	Electromyogram (EMG)
Application scenarios	Age-friendly, smart sofa	Multimedia tools	Encoder training	Music generation	Deepen research	Emotion regulation

a EEG: electroencephalogram.

b ERP: event-related potential.

c EMER-CL: embedding-based music emotion recognition using composite loss.

These studies all aim to expand the possibilities of music emotion recognition from different perspectives, thus enabling mutual supplementation and validation. Through comparative and correlational analysis with other literature, this study demonstrates significant differentiation and innovation in several aspects. First, unlike previous studies that mainly targeted the mixed-age groups [112,113], this study specifically focuses on the older adult population, aiming to address the specific emotional regulation needs of the older adults in smart home environments. Methodologically, this study obtained the physiological responses of older adult participants through EEG and ERP analyses and established an emotional state mapping in combination with musical parameters. The MF-AWI model proposed in this study integrates multimodal data and health-promoting HCI, which differs from existing multimodal emotion recognition studies in terms of model framework. Examples include the rhythm prediction model by Wang et al [114], or the experiments on young and mixed-age groups by Sutcliffe et al [115] and Vieillard and Gilet [116]. The proposed model not only considers music and emotion recognition but also emphasizes individual adaptability in home interaction scenarios. Furthermore, numerous studies have demonstrated that multimodal fusion methods outperform single-modality approaches in emotion recognition tasks. For instance, when combining EEG with facial expressions for emotion classification, multimodal fusion consistently achieves higher accuracy than unimodal methods [117]. Therefore, the MF-AWI model incorporates emotional gradients and health-promoting thresholds in feature fusion, enabling personalized emotional feedback, and its preliminary validation in the intelligent home sofa scenario further highlights the potential of multimodal emotion recognition and regulation for practical adaptability and operability.

### Limitations

This study has several limitations. First, regarding the participant population, the sample was restricted to older adults aged 60‐75 years. Future research should further refine user needs and personal habits by examining behavioral habits and individual preferences, as well as potential gender differences in emotional responses and interaction behaviors. Second, all participants in this study were healthy older adults, as the primary aim was to establish a foundational model for music–emotion interaction within smart home systems. However, individuals with cognitive decline, dementia, or other impairments, who represent key potential beneficiaries of such systems, were not included [118]. Since cognitive processing and emotional responses may differ between healthy and cognitively impaired older adult populations, the generalizability of the findings remains to be verified in special older adult groups. Future studies should therefore expand the sample to include older adults with varying levels of cognitive function to comprehensively assess the applicability and effectiveness of the music interaction module across diverse user groups. Comparative studies between groups with different health statuses are also recommended to better understand how cognitive conditions influence emotional and behavioral responses to such interactive systems.

At the model and system level, this study primarily focused on using experimental data to validate the feasibility of the multimodal emotion recognition and adaptive music-matching mechanisms within the MF-AWI model. The study is positioned as a mechanism-level validation. In addition, the CNN–DeepFace emotion recognition component in this study mainly served to verify the feasibility of integrating visual emotion detection into the interactive music system, rather than to develop a fully trained and optimized recognition model. Consequently, systematic quantitative evaluations of the multimodal emotion recognition module and the adaptive feedback module, such as accuracy and *F*_1_-score, have not yet been conducted, and their performance cannot be directly compared with existing EEG-based, multimodal, or single-modality emotion recognition models. Future work will involve large-scale experiments and cross-context validation, complemented by usability testing and subjective satisfaction assessments, to comprehensively evaluate the model’s effectiveness in real-world application scenarios. The model can also be further trained and optimized using larger and more diverse datasets of older adult users to enhance recognition accuracy and generalizability. In addition, standardized performance metrics will be applied to compare multimodal fusion methods with unimodal approaches in home-based environments, thereby further validating the practical benefits of the model in emotion recognition tasks. Finally, regarding statistical analysis, post hoc pairwise comparisons were performed using the LSD method following significant ANOVA results. As this study primarily focused on examining neural activity differences elicited by different musical stimuli, the LSD method provides high sensitivity for detecting effects. However, no further strict multiple comparison corrections were applied, which may increase the risk of type I errors. Future studies will consider using more rigorous correction methods, such as Bonferroni or false discovery rate adjustments, to verify the robustness of the results.

From an application and technical perspective, this study also has certain limitations. The experimental validation was carried out using a leisure sofa as the primary prototype. Future work could integrate multiple types of sensors to collect multichannel physiological signals, thereby improving emotion recognition accuracy and response speed. Moreover, EEG signal acquisition remains susceptible to environmental noise, poor electrode contact, and participant movement. Subsequent studies should focus on improving signal processing algorithms and denoising strategies, enhancing model generalization, and conducting long-term validation in real-world smart home environments to ensure system reliability and sustained usability and user experience.

Finally, from a social and cultural perspective, this study did not deeply address issues related to cross-cultural contexts or the socio-psychological effects of such systems among older adults. Although the system design and experiments were intended to promote user well-being, future interdisciplinary research should consider cultural diversity and social inclusiveness when evaluating and optimizing emotional smart products for the older adult population.

### Conclusions

The research object of this paper is explicitly for the older adult users, and it aims to solve the problems related to the emotions of older adult users in their home environment. At the same time, the mapping relationship between music parameters and emotional state is constructed. In conjunction with experimental and physiological data analysis, several aspects are considered, and it is concluded that A1 stimulus materials can soothe the emotions of older adult users. In terms of research methodology, this paper uses EEG and ERP analysis, and the application scene is a smart sofa for older adults. It is compatible with the research object and has a certain degree of practicality. At the same time, based on this, the music emotion interaction module in the multimodal HCI system of the health care sofa is set up, and it provides health care thresholds and theoretical support for the health care sofa.

## Abbreviations

CNN: convolutional neural network
EEG: electroencephalogram
ERP: event-related potential
HCI: human-computer interaction
LSD: least significant difference
MF-AWI: Multimodal Fusion-Adaptive Wellness Interaction
VA: Valence-Arousal
